# Boer and British Sanitation

**Published:** 1900-08-11

**Authors:** 


### BOER AND BRITISH SANITATION.

Dr. Herbert Caiger,[1] writing from Burghersdorp gives various details about the medical and sanitary arrangements among the troops at that place. He says that the absence of proper sanitary regulations in the Boer laager at Stormberg was a disgrace to any nation that pretended to civilisation. Dead animals and the remains of slaughtered sheep lying about unburied caused a terrible stench. In the offices and waiting-rooms at the railway stations all sorts of refuse were allowed to accumulate, and in some instances dead horses were left behind in the railway offices. At the same time, he says, the sanitary arrangements for our troops who were stationed in or passed through the district were not free from reproach. At Bethulie Bridge camp the stench along the river bank, and for some considerable distance from the river, was bad, and the same thing was noticeable at Sterkstoom camp; while at Burghersdorp, when large numbers of troops were passing through, the station yard was sometimes in a disgusting state. He very properly says that these things ought not to be, and that there is still less excuse in the camps. "Either the sanitary arrangements must have been at fault, or the discipline for their proper enforcement must have been lax."

[1] Lancet, July 28.

In a paper recently communicated to the Royal Society by Lord Lister, Dr. Allen Macfadyen and Mr. Sydney Rowland describe some experiments which they have been making on the influence of the temperature of liquid hydrogen upon bacteria. They had previously shown that the temperature of liquid air has no appreciable effect upon the vitality of micro-organisms, even when they are exposed to it for a week, and they now show that a variety of micro-organisms, including among many others the bacillus typhosus, the bacillus diphtheriæ, anthracis, and coli communis, together with a sarcina and a yeast, could be exposed to a temperature of 252 deg. C. for 10 hours without producing any appreciable effect upon their vitality. The bearing of these observations upon many moot questions is obvious. It can hardly be imagined that at such temperatures vital processes persist. We are almost forced to believe that for the time everything is in abeyance. Yet, on removal of the inhibitory condition, vital phenomena show themselves afresh and unimpaired—which is interesting.

